# Investigating the Feasibility of Using a Wearable Device to Measure Physiologic Health Data in Emergency Nurses and Residents: Observational Cohort Study

**DOI:** 10.2196/51569

**Published:** 2024-02-22

**Authors:** Anish K Agarwal, Rachel Gonzales, Kevin Scott, Raina Merchant

**Affiliations:** 1 Perelman School of Medicine University of Pennsylvania Philadelphia, PA United States; 2 Department of Emergency Medicine University of Pennsylvania Philadelphia, PA United States; 3 Center for Health Care Transformation and Innovation Penn Medicine Philadelphia, PA United States

**Keywords:** digital health, emergency medicine training, wearable devices, burnout, mobile health, feasibility, wearable device, wearable, physiologic health data, nurse, resident, emergency department, acceptability, well-being

## Abstract

**Background:**

Emergency departments play a pivotal role in the US health care system, with high use rates and inherent stress placed on patients, patient care, and clinicians. The impact of the emergency department environment on the health and well-being of emergency residents and nurses can be seen in worsening rates of burnout and cardiovascular health. Research on clinician health has historically been completed outside of clinical areas and not personalized to the individual. The expansion of digital technology, specifically wearable devices, may enhance the ability to understand how health care environments impact clinicians.

**Objective:**

The primary objective of this pilot study was to assess the feasibility and acceptability of using wearable devices to measure and record physiologic data from emergency nurses and resident physicians. Understanding strategies that are accepted and used by clinicians is critical prior to launching larger investigations aimed at improving outcomes.

**Methods:**

This was a longitudinal pilot study conducted at an academic, urban, level 1 trauma center. A total of 20 participants, including emergency medicine resident physicians and nurses, were equipped with a wearable device (WHOOP band) and access to a mobile health platform for 6 weeks. Baseline surveys assessed burnout, mental health, and expectations of the device and experience. Participants provided open-ended feedback on the device and platform, contributing to the assessment of acceptance, adoption, and use of the wearable device. Secondary measures explored early signs and variations in heart rate variability, sleep, recovery, burnout, and mental health assessments.

**Results:**

Of the 20 participants, 10 consistently used the wearable device. Feedback highlighted varying experiences with the device, with a preference for more common wearables like the Apple Watch or Fitbit. Resident physicians demonstrated higher engagement with the device and platform as compared with nurses. Baseline mental health assessments indicated mild anxiety and depressive symptoms among participants. The Professional Fulfillment Index revealed low professional fulfillment, moderate workplace exhaustion, and interpersonal disengagement.

**Conclusions:**

This pilot study underscores the potential of wearable devices in monitoring emergency clinicians’ physiologic data but reveals challenges related to device preferences and engagement. The key takeaway is the necessity to optimize device and platform design for clinician use. Larger, randomized trials are recommended to further explore and refine strategies for leveraging wearable technology to support the well-being of the emergency workforce.

## Introduction

Emergency departments (EDs) are a critical and frequently used resource within the US health care infrastructure (1 in 5 adults are treated in an ED annually) [1,2]. EDs are stressful environments [3-6]. Research has demonstrated the negative impact of the physical ED environment on patients [7-10], yet less is known about the effects on the health and well-being of the clinicians working within these EDs. Burnout and cardiovascular (CV) health remain threats to ED clinicians, their careers, and patient care [11-14]. Research on clinician health and burnout has historically been limited by retrospective studies which are often conducted outside of work. The rapid growth of digital technology, such as wearable devices and remote monitoring [15-17], offers new opportunities and challenges to investigate clinician health within health care environments and spaces.

Multiple occupational hazards have been attributed to the practice of emergency medicine (EM) [18], EM clinicians have a 20% higher morbidity due to coronary artery disease, motor vehicle accidents, and impaired reproductive health [19-22]. Clinicians working night shifts, an essential practice in EM, have less restorative sleep, elevated blood pressure, and lower heart rate variability (HRV) [19,23-26]. Before the pandemic, the prevalence of stress, exhaustion, and burnout was alarmingly high in EM [27,28]. COVID-19 worsened these factors, resulting in workforce depletion, and making this an urgent and critical area of focus underscored by the Surgeon General and National Academy of Medicine [14,29].

A gap exists in understanding how clinicians identify and prioritize their health within the workplace. High-performance athletics provides a potential analogous framework whereby athletes track physiologic data (HRV, physical activity, and sleep) to guide their daily performance. There is an unrealized opportunity space for clinicians to understand and enhance care delivery and career longevity (reduce burnout and CV disease). Technological advancements provide a potentially unobtrusive and personalized method to collect individual data using wearable devices. Wearable device use has grown in popularity, with over 30% of Americans reporting they can obtain device ownership [30]. These wearable devices are typically wrist-worn and provide methods to measure health data such as step count, heart rate, and sleep [15]. What is less known, is if these devices and associated platforms are appealing to clinicians and can provide actionable insights to help inform strategies to support the workforce.

The objective of this study was to pilot test and evaluate the feasibility and acceptability of a wearable device and associated platform to measure and record emergency nurse and resident physician physiologic measures while they provide emergency care. This was a pilot study, investigating early barriers and facilitators to using these devices within health care settings for emergency nurses and residents.

## Methods

### Overview

Eligible EM resident physicians and emergency nurses included those providing 20 or more hours of patient care per week, having regular access to a smartphone, and providing consent to where a wrist-worn wearable device (WHOOP band [31]). Participants were recruited via email, completed informed consent, and were given a wrist-worn wearable device. Consenting participants completed a baseline survey assessing burnout and mental health (depression and anxiety), asked about their expectations of the study, and followed for 6 weeks. Validated instruments included the Patient Health Questionnaire (PHQ-8), General Anxiety Disorder (GAD-7), and the Professional Fulfillment Index (PFI) [32-36]. Over the 6 weeks, patients were also given access to a web-based platform that allowed participants to see their own physiologic data, access basic coaching videos, and connect to other users on the platform (Arena Strive [37]). At the completion of 6 weeks, participants were asked to complete a final survey exploring the feasibility and acceptability of the approach. Participants were also asked to provide free text commentary on the general approach and the specific device. The primary outcomes were use of the wearable device, acceptance of the device, and adoption of the device. Secondary measures included burnout, mental health symptoms, and physiologic measures recorded by the device including HRV and sleep.

### Ethical Considerations

This was a longitudinal pilot feasibility study conducted at an urban, academic, level 1 trauma center in the northeastern United States. This study was reviewed and approved by the University of Pennsylvania Institutional Review Board (850371). All eligible participants completed informed consent forms and were informed that all data would be deidentified and aggregated for analysis. Participants received the wearable device at no cost and could keep the device following the completion of the study.

### Data Analysis

Analysis was conducted using Stata SE (version 18; StataCorp). Descriptive statistics were used to summarize participant demographics and well-being survey results which included the PHQ-8, GAD-7, and the PFI. Single-sample 2-tailed *t* tests were used to investigate exploratory differences in physiologic measures.

## Results

This was a pilot feasibility study and thus was not powered to detect individual health outcomes. A total of 20 participants were enrolled (13/20, 65% were female), 12 were EM resident physicians and 8 were emergency nurses. Of the 20 participants, 10 participants routinely wore the wearable device (6 resident physicians and 4 nurses).

Participants completing baseline mental health assessments reported mild anxiety as measured by the GAD-7 (mean score 5.07, SD 3.7), with 85% (n=17) reporting minimal or mild anxiety. Participants also reported mild depressive symptoms as measured by the PHQ-8 (mean 5.73, SD 2.9), with half reporting mild depressive symptoms. Participants completed the PFI to evaluate burnout and fulfillment. Individuals reported low professional fulfillment (mean 49.4, SD 16.9) moderate workplace exhaustion (mean 57.1, SD 24.4), and moderate interpersonal disengagement (mean 44.7, SD 20.1). Participants were asked via survey to comment on their early thoughts and goals with the pilot and the device. Notable themes emerged reflecting (1) technological features (eg, seeking a device with a watch face), (2) ways to integrate data into their personal lives and clinical roles, and (3) increasing self-awareness of the objective measures of stress related to clinical care.

Among participants who used the band consistently for 6 weeks, variation existed in their experience with the wearable and the data it generated. None of the participants were very likely to recommend the device to others. Two participants found the data interaction helpful and useful and overall, none commented on the platform being easy to use. When asked specifically, participants noted the band to be obtrusive given its lack of daily use features (eg, watch face and activity data) and odd charging mechanics. Several participants did comment positively that the data output and data generated was useful and empowering but needed to be collected using a more user-friendly design such as the more commonly used Apple Watch or FitBit. Participants sought the same application using these devices and expressed enthusiasm for those.

**Figure 1:**
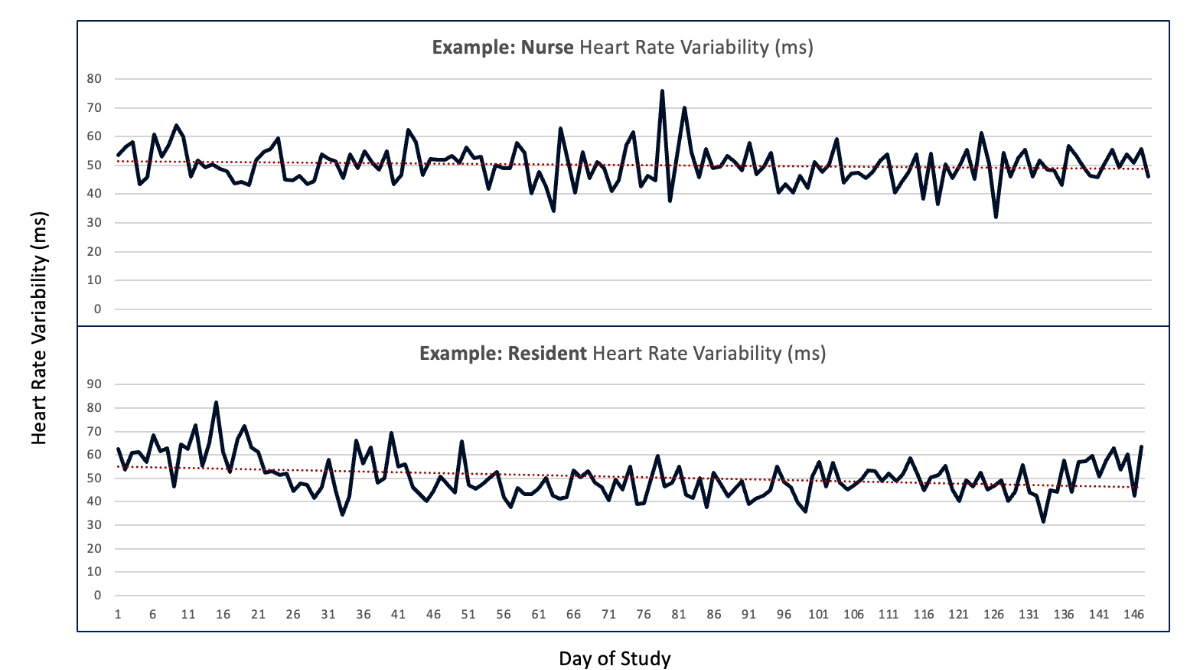
An example, exploratory data synopsis at the participant level of heart rate variability (HRV) over the study period. The top pane depicts the HRV variation for an emergency nurse and the bottom for an emergency resident. This example snapshot can be viewed by participants on the mobile platform.

**Figure 2:**
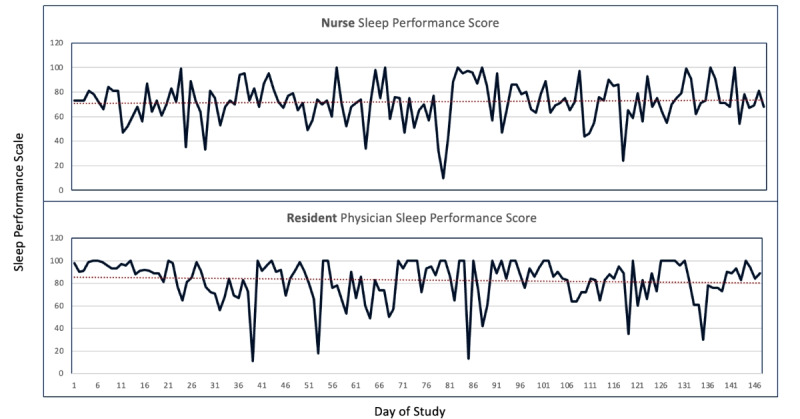
An example, exploratory data synopsis at the participant level of sleep performance over the study period. The top pane depicts sleep performance variation for an emergency nurse and the bottom for an emergency resident. This example snapshot can be viewed by participants on the mobile platform. The sleep performance indicator is a composite measure proprietary to the device platform.

Of the 10 users who routinely used the device, we saw early variation in physiologic measures related to HRV, stress, and sleep (Table 1; Figures 1 and 2). While statistically significant differences are identified here, this remained a pilot study in feasibility, user input, and data collection methods. Early insights from these data suggest differences across roles between resident physicians and nurses, as well as across sex.

**Table 1:** Participant wearable device data.

	Overall (n=10); mean (SD)	Residents (n=6); mean (SD)	Nurses (n=4); mean (SD)	*P* value	Women (n=4); mean (SD)	Men (n=6); mean (SD)	*P* value
Days used	72.6 (49.7)	72.8 (46.9)	72.3 (61.1)	.99	50.5 (38.7)	87.3 (53.8)	.28
Recovery score	59.5 (22.9)	60.7 (22.6)	57.7 (23.1)	.08	55.7 (25.7)	61.0 (21.5)	.01
Resting heart rate	58.8 (7.4)	58.5 (5.6)	59.3 (9.4)	.16	63.3 (7.2)	57.1 (6.7)	<.001
Heart rate variability (ms)	50.4 (17.8)	47.3 (12.8)	55.1 (22.8)	<.001	55.8 (29.1)	48.3 (10.0)	<.001
Sleep performance score	77.1 (19.1)	78.3 (19.4)	75.1 (18.6)	.03	78.2 (21.2)	76.6 (18.3)	.30
Sleep disturbances	10.1 (5.8)	10.1 (5.5)	10.0 (6.3)	.82	13.1 (6.1)	8.9 (5.3)	<.001

## Discussion

### Principal Findings

The physical and mental toll facing clinicians working in the ED continues to grow. Emergency nurses and resident physicians face a number of challenges impacting their health including, but not limited to, ED crowding, boarding, workplace violence, shifting schedules, and rising patient acuity. In the wake of the COVID-19 pandemic, an emphasis on supporting the workforce remains a priority. The evolving landscape of digital technology, including wearable devices, offers new opportunities for individuals to monitor their own health and potentially proactively identify physical or mental strain. This pilot study begins to examine if and how wearable devices can be used for emergency clinicians.

First, we found mixed enthusiasm for this approach given low interest in completing exit surveys and ongoing data interaction. It appears from this early feasibility study that resident physicians may be more engaged with this strategy and data collection method. Nurses in this pilot tended to be less engaged with the data tracking and follow-up mechanisms. Resident physicians were, in general, more enthusiastic before, during, and after the period ended. Though small in size, this pilot study does shed some initial interest from emergency clinicians and understanding key physiologic metrics such as sleep and physical activity. Key data insights remain physical activity as measured by step count, amount and quality of sleep, and HRV, which is an established physiologic measure relating to CV health [38]. The next steps to build on this pilot study included bringing it to a larger scale and designing it for nurse and physician preferences which we have learned to date.

The key finding of this pilot study is that the type of device and platform must be optimized for clinician use. In this study, we used a high-performance athletics device, which is designed primarily for physiologic measures [39]. This device does not have some traditional features that the average person may be accustomed to including a clock and the ability to send or receive messages. More traditional and popular bands such as the Apple Watch or Fitbit offer participants data tracking with features such as message-sending capability and a watch. These features are important when designing for scale, but were not previously known until we pilot-tested it, as other studies in more controlled environments have used the same device [39,40], underscoring the need to optimize design over technological capacity. The high-performance athletic band used in this pilot study offered a longer battery life and the ability to charge while wearing the device, which seemed less important to participants in this study. Future work needs to leverage the existing devices that clinicians wear in their everyday lives and incorporate those devices into this approach.

Finally, these devices and remote surveys highlight the persistent and real variation in professional disengagement, exhaustion, and burnout. In this small cohort, we do not identify significant amounts of anxiety or depression in screening assessments. We do note some variation and physiologic measures across resin physicians and nurses as well as differences across individuals who identify as male vs female. These differences though statistically significant, represent only a small sample size, and follow-up studies need to be scaled at larger populations. Specific interests should investigate physiologic measures such as heart rate, HRV, and sleep.

### Limitations

This study has several limitations. This was a single-center pilot study and had a small sample size, which was intentional by design. We see glimpses toward mechanisms to optimize digital technology and workforce sustainment. The signals identified here represent only early pilot findings, and inherently there is also selection bias in individuals who opted to wear the band and complete surveys. Less is known about individuals who do not want to be part of this pilot, and future studies will need to be larger, randomized, and for a longer duration. Nonetheless, the study is among the first to begin to investigate the feasibility of using digital technology to support emergency physicians and nurses by helping them identify physiologic variations in their own health. The study represents the pilot beginnings to help identify proactive and much-needed new methods to mitigate strain that is related to physical and mental health.

### Conclusion

This pilot study of emergency nurses and resident physicians investigating wearable devices to capture physiologic data from a cohort represents early signals toward feasible and acceptable programs. This pilot study identifies opportunities and interest in these mechanisms and a need to leverage more consumer-facing and potentially less sophisticated wearable devices for emergency clinicians. These methods can be further explored and larger, randomized trials can be conducted to investigate these strategies and how we support the workforce.

## Abbreviations

CV: cardiovascular
ED: emergency department
EM: emergency medicine
GAD-7: General Anxiety Disorder
HRV: heart rate variability
PFI: Professional Fulfillment Index
PHQ-8: Patient Health Questionnaire

## Data Availability

The deidentified data sets generated or analyzed during this study are available from the corresponding author on reasonable request.
